# PI3K Signaling in Dendritic Cells Aggravates DSS-Induced Colitis

**DOI:** 10.3389/fimmu.2022.695576

**Published:** 2022-04-19

**Authors:** Mario Kuttke, Dominika Hromadová, Ceren Yildirim, Julia S. Brunner, Andrea Vogel, Hannah Paar, Sophie Peters, Maria Weber, Melanie Hofmann, Martina Kerndl, Markus Kieler, Hannes Datler, Laszlo Musiejovsky, Manuel Salzmann, Michaela Lang, Klara Soukup, Angela Halfmann, Omar Sharif, Gernot Schabbauer

**Affiliations:** ^1^ Institute for Vascular Biology and Thrombosis Research, Center for Physiology and Pharmacology, Medical University of Vienna, Vienna, Austria; ^2^ Department of Gastroenterology and Hepatology, Internal Medicine III, Medical University of Vienna, Vienna, Austria; ^3^ St. Anna Children’s Cancer Research Institute, Vienna, Austria

**Keywords:** PI3K, PTEN, dendritic cells, DSS-induced colitis, Interleukin-6, Th1-response

## Abstract

Aberrant innate immune responses to the gut microbiota are causally involved in the pathogenesis of inflammatory bowel diseases (IBD). The exact triggers and main signaling pathways activating innate immune cells and how they modulate adaptive immunity in IBD is still not completely understood. Here, we report that the PI3K/PTEN signaling pathway in dendritic cells enhances IL-6 production in a model of DSS-induced colitis. This results in exacerbated Th1 cell responses and increased mortality in DC-specific PTEN knockout (PTEN^ΔDC^) animals. Depletion of the gut microbiota using antibiotics as well as blocking IL-6R signaling rescued mortality in PTEN^ΔDC^ mice, whereas adoptive transfer of Flt3L-derived PTEN^-/-^ DCs into WT recipients exacerbated DSS-induced colitis and increased mortality. Taken together, we show that the PI3K signaling pathway in dendritic cells contributes to disease pathology by promoting IL-6 mediated Th1 responses.

## Introduction

Dendritic cells (DC) constitute an important cellular part of the immune system, bridging innate and adaptive immune responses. In the gut they mediate tolerance to food antigens, regulate responses to the commensal microbiota and induce immunity against pathogens. Apart from genetic factors regulating barrier function, autophagy and endoplasmic reticulum (ER) stress in the epithelium, the microbiota themselves as well as aberrant and deregulated immune responses directed against gut microbiota were shown to be causally involved in the development of inflammatory bowel disease [IBD; summarized in (1, 2)]. IBD comprises two main subtypes, Crohn’s disease (CD) and ulcerative colitis (UC). Whereas in the former the whole gastrointestinal tract might be affected by a Th1 mediated response, the latter is usually confined to the colon and is mainly Th2 driven (1–3).

The role of DCs in the development and progression of IBD is still not completely elucidated. In animal models of colitis, diphtheria toxin-mediated depletion of DCs has produced conflicting results, reporting protection (4) or exacerbation of disease (5). Systemic and gut DCs depend on FMS-like tyrosine kinase 3 ligand (Flt3L) for their development (2). Binding of this hematopoietin to its receptor, Flt3 (CD135), leads to downstream activation of the PI3K/AKT/mTOR pathway (6). In addition, activation of PI3K/AKT/mTOR signaling has been shown to downregulate inflammatory responses in mature macrophages as well as DCs and induce regulatory programs highlighting the importance of this pathway during DC development and in regulating the function of innate immune cells (7–12). The PI3K/AKT/mTOR pathway is counteracted by the phosphatase PTEN (phosphatase and tensin homologue deleted on chromosome 10) and DC-specific knock-out of PTEN has been shown to result in increased PI3K activity in DCs (6). Still, the exact role of PI3K signaling in the regulation of DC function during colitis is ill defined.

We could recently show that activation of myeloid PI3K signaling drives immunosuppressive CD8α^+^DCs that impair antitumor T cell responses in a model of colitis-associated colorectal cancer (13). To further elucidate the role of this signaling pathway in DCs during colitis and colitis-driven cancer, we generated DC-specific PTEN-deficient (PTEN^ΔDC^) mice. Quite unexpectedly, these mice showed a high incidence of mortality in both models which occurred very early and coincided with increased IL-6 serum levels and elevated Th1 cell responses in the initial inflammatory phase, contrasting the reported anti-inflammatory actions of PI3K signaling. We could show that the high mortality in these animals can be rescued with antibiotics treatment or blocking IL-6R signaling, which reduced Th1 responses. Our work highlights the importance of the PI3K signaling pathway in modulating DC responses during intestinal inflammation, suggesting the possibility of blocking this pathway in patients with DC driven inflammatory diseases.

## Materials and Methods

### Mice

Floxed PTEN mice (Pten^tm2Mak^, MGI:2182005) were a kind gift from Tak W. Mak and were crossed with CD11c cre (Tg(Itgax-cre)1-1Reiz, MGI:3763248) to generate PTEN^loxP/loxP^CD11cCre^+/-^ (PTEN^ΔDC^) or crossed with LysM cre (8) to generate PTEN^loxP/loxP^LysMCre^+/-^ (PTEN^Δmye^) in house. Ovalbumin-specific TCR transgenic OTII mice (B6.Cg-Tg(TcraTcrb)425Cbn/J, MGI:2174541) were purchased from Jackson Laboratory and bred in house. C57BL/6J mice were bred at the Department for Biomedical Research of the Medical University of Vienna. All mice were backcrossed to C57BL/6 background for at least 10 generations and housed under SPF conditions. All animal experiments were performed in accordance with the regulations of the Ethics Committee of the Medical University of Vienna and approved by the Austrian Federal Ministry for Education, Science and Research (BMWFW-66.009/0055-II/3B/2014 & 66.009/0317-WF/V/3b/2015). Conditional PTEN-deficient mice were genotyped in a direct PCR reaction (GoTaq Polymerase; Promega) using tissue lysates as templates. The following primers were used for genotyping: cre-for: 5’-TCGCGATTATCTTCTATATCTTCA-3’; cre-rev: 5’-GCTCGACCAGTTTAGTTACCC-3’; fl-PTEN-for: 5’-CTCCTCTACTCCATTCTTCCC-3’; fl-PTEN-rev: 5’-ACTCCCACCAATGAACAAAC-3’.

### DSS-Induced Colitis and Colitis-Associated Colon Cancer Induction

DSS-colitis was induced by supplementation of drinking water with 2-4% DSS (36-50kDa, MP Biomedicals), depending on experimental setup and DSS batch. Colitis-associated colon cancer (CAC) was induced as reported previously (13). In brief, 8-12 week old mice were injected with 12.5mg/kg azoxymethane (AOM, Sigma-Aldrich) i.p. followed by supplying 2.5% DSS in drinking water 6 days post AOM injection. After 6 days of DSS supplementation, mice were switched to drinking water for 15 days. This was repeated for a total of 3x DSS/water cycles. Mice were sacrificed on days 85-90 after AOM injection. In experiments using antibiotics treatment mice were s.c. or i.p. injected with 7mg/kg enrofloxacin (Baytril^®^, Bayer) daily, starting on the first day of DSS-supplement. In experiments using IL-6R blockade, mice were treated with 500µg IL-6R mAb (clone 15A7, BioXCell) or isotype control (clone LTF-2, BioXCell) twice a week. Mice were monitored and weighed daily. Cytokines in plasma of mice were detected by standard ELISA (R&D) according to manufacturer’s instructions.

### Single Cell Preparation

Single cells from spleens and mesenteric lymph nodes were obtained by passing the tissues through 70µm cell strainers into RPMI medium (containing 10% FBS, penicillin, streptomycin and fungizone). Single cell suspensions of splenocytes were further subjected to erythrocyte lysis (150mM NH_4_Cl, 10mM KHCO_3_, 0.1mM Na_2_EDTA). Cells were either counted by flow cytometry (Beckman Coulter Cytoflex S) or by an automated cell counter (Bio-Rad TC10). Colons were cleared of content, rinsed with PBS and incubated with 5mM EDTA for 2x 20min to obtain epithelial cells, followed by enzymatic digestion with 10mg collagenase VIII (Sigma) and 5mg DNAseI (Worthington) in PBS per colon to isolate cells from colon lamina propria (CLP).

### Generation of Bone Marrow-Derived Dendritic Cells

Bone marrow from tibiae and femurs was flushed with PBS and subjected to erythrocyte lysis. Cells were adjusted to 10^6^ cells/ml in fully supplemented RPMI-1640. Medium was supplemented with 100ng/ml Flt3L (eBioscience) to generate FL-DCs or 20ng/ml GM-CSF (R&D) and 5ng/ml IL-4 (Peprotech) to generate G4-DCs. Fresh medium (50% of initial volume) supplemented with Flt3L, or GM-CSF/IL-4 was added after 3 days. Non-adherent cells in GM-CSF/IL-4 culture or total Flt3L cultured cells were harvested on day 7 and 10^6^ cells were adoptively transferred i.v. into C57BL/6J recipients. FL-DCs were further stimulated for 24 h with poly(I:C)-HMW (1µg/ml, *Invivogen*), poly(I:C)-LMW (1µg/ml, *Invivogen*), LPS (10ng/ml; *Invivogen*), imiquimod (IMQ, 1µg/ml, *Invivogen*), CpG-ODN1585 (CpG-A, 1µM, *Invivogen*), CpG-ODN1668 (CpG-B, 1µM, *Invivogen*) and supernatants were analyzed for IL-6 protein using flow cytometry (Legendplex beads, Biolegend) or ELISA (R&D).

### *Ex Vivo* T Cell Responses

To study T cell responses ex vivo, splenocytes were generated from colitic mice and 0.2x10^6^ cells were stimulated with activating CD3 and CD28 (1µg/ml each, eBioscience) for 3 days in fully supplemented RPMI-1640 medium. For co-culture experiments with OTII T cells, splenic CD11c^+^ cells from WT and PTEN^ΔDC^ were enriched using magnetic beads (Miltenyi Biotec) and co-incubated with CD4^+^ enriched splenic OTII T cells (DC:T cell ratio 1:4) in the presence of 100ng/ml LPS and 50µg/ml ovalbumin in fully supplemented RPMI-1640 medium. IFNγ levels in supernatants were analyzed by ELISA (R&D).

### Flow Cytometry

For flow cytometry analysis, single cell suspensions from spleens, mesenteric lymph nodes and colon LP and epithelium were stained using the following antibodies: CD45.2-APC-eFluor780 (clone 104, eBioscience, 47-0454-82), CD3-PE-CF594 (clone 145-2C11, BD Biosciences, 562286), CD4-PerCP-Cy5.5 (clone RM4-5, BD Biosciences, 550954), CD4-PerCP-eFluor710 (clone RM4-5, eBioscience, 46-0042-82), CD8a-PE (clone 53-6.7, eBioscience, 12-0081-82), CD8α-Brilliant Violet 510 (clone 53-6.7, BioLegend, Brilliant Violet™ products are trademark of Sirigen Group Ltd., 100752), B220-Alexa700 (clone RA3-6B2, eBioscience, 56-0452-82), CD11b-APC (clone M1/70, eBioscience, 17-0112-8), CD11c-eFluor450 (clone N418, eBioscience, 48-0114-82), MHCII-FITC (clone M5/114.15.2, eBioscience, 11-5321-82), CD103-Brilliant Violet 510 (clone 2E7, Biolegend, 121423), CD80-PE-CF594 (clone 16-10A1, BD Biosciences, 562504), CD86-BV650 (clone GL-1, BioLegend, 105035), CD80-FITC (clone 16-10A1, Tonbo Bioscience, 35-0801), B220-PE-Cy5 (clone RA3-6B2, Tonbo Bioscience, 55-0452), CD40-APC (clone FGK45, Tonbo Bioscience, 20-8050), MHCII-eF450 (clone M5/114.15.2, eBioscience, 48-5321-82), CD86-BV605 (clone GL-1, BioLegend, 105037), CD45.2-BV650 (clone 104, BioLegend, 109836), CD11b-PE-Cy7 (clone M1/70, eBioscience, 25-0112), SiglecH-FITC (clone 551, BioLegend, 129604), Bst2-PerCP-eF710 (clone 927, eBioscience, 46-3172-82), B220-APC (clone RA3-6B2, Tonbo Bioscience, -20-0452), Ly6G-AF700 (clone 1A8, BioLegend, 127622), CD11c-APC-eF780 (clone N418, eBioscience, 47-0114), CD11b-PB (clone M1/70, Invitrogen, RM2828), MHCII-PE (clone M5/114.15.2, Tonbo Bioscience, 50-5321), Ly6C-PE-Cy7 (clone HK1.4, BioLegend, 128018), SYTOX AADvanced/7AAD (Invitrogen, S10274), CD4-FITC (clone GK1.5, Tonbo Bioscience, 35-0041), NK1.1-APC (clone PK136, Tonbo Bioscience, 20-5941), CD3-rF710 (clone 17A2, Tonbo Bioscience, 80-0032), CD19-BV510 (clone 6D5, BioLegend, 115545), CD8a-PE (clone 53-6.7, Tonbo Bioscience, 50-0081). Flow cytometry acquisition was performed on a LSR Fortessa flow cytometer (BD Biosciences) or a Cytoflex S flow cytometer (Beckman Coulter), Data were analyzed with FlowJo software version 10 (Treestar). For intracellular cytokine production analysis, cells from spleens and mesenteric lymph nodes were isolated and re-stimulated with anti-CD3/CD28 dynabeads (Life Technologies) or PMA/Ionomycin (Sigma Aldrich) together with Golgi-Stop (BD Biosciences) for 4 hours. Cytokines stainings were performed by using eBioscience’s IC Fixation & Permeabilization Buffer kit following the manufacturer’s instructions with the following antibodies: IFNγ – PE (clone XMG1.2, eBioscience, 12-7311), IFNγ – PE (clone XMG1.2, BioLegend, 505808), IL-17 – PE-Cy7 (clone ebio17B7, eBioscience, 25-7177), IL-2 – eFlour 450 (clone JES6-5H4, eBioscience, 48-7021), IL-10 – APC (clone JES5-16E3, eBioscience, 17-7101). For Foxp3 intracellular staining, cells from spleens and mesenteric lymph nodes were isolated and stained for surface markers followed by fixation and permeabilization using Foxp3/Transcription Factor Staining Buffer Kit (Tonbo Biosciences, TNB-0607) and incubation with Foxp3-APC antibody (clone 3G3, Tonbo Biosciences, 20-5773) according to manufacturer´s protocol.

### Histology

Mice were sacrificed, colons were removed, rinsed with PBS and formalin-fixed overnight. Tissues were further dehydrated and paraffin-embedded as “Swiss rolls”. 5µm sections were used for H&E staining using standard protocols. Stained sections were scanned on an Olympus VS-BX61 microscope.

### Colonoscopy and Scoring

Before colonoscopy, mice were fasted for 24 h, receiving a rehydration solution (13.5 g/l glucose, 2.9 g/l trisodium citrate dihydrate, 2.6 g/l sodium chloride and 1.5 g/l potassium chloride) supplemented with 34.5 g/l polyethylene glycol (molecular weight 35000 g/mol). For colonoscopy, mice were anesthetized. During insertion and withdrawal of the colonoscope (Karl Storz, Tuttlingen, Germany), the grade of inflammation was scored using following parameters: hyperplasia in the distal or proximal colon (grade 0-3), presence of a stricture (0–1), fibrin deposition (0–1), vulnerability of the mucosa (0–1), bleeding (0–1), and visibility of the vasculature (0–3). Videos of the colonoscopies were recorded.

### Measurement of Gut Permeability

Gut permeability was assessed as previously described (14). In brief, mice were starved overnight. On the following day, mice were weighed and orally gavaged with 440mg/kg FITC-dextran (FD4, Sigma Aldrich) in PBS. Blood was drawn 4 hours later, and serum concentration of FITC-dextran was quantified by using a FITC-dextran standard curve diluted in PBS.

### RT-qPCR

RNA was isolated from colon lamina propria single cell suspension using Trifast (PeqLab) and reverse-transcribed into cDNA using High Capacity cDNA Reverse Transcription Kit (Fermentas) according to manufacturer’s protocols. Expression of mRNA was measured using FastSybrGreen Mix (Applied Biosystems) in a StepOne RT-PCR 7500 system (Applied Biosystems). Ct-values of target genes were normalized to mHPRT and expressed as fold mean WT control using efficiencies calculated using LinReqPCR (15) for each amplification. The following primers were used:

hprt: for: CGCAGTCCCAGCGTCGTG; rev: CCATCTCCTTCATGACATCTCGAG;

il10: for: AGCTGAAGACCCTCAGGATG; rev: TGGCCTTGTAGACACCTTGG;

ifng: for: TGAGCTCATTGAATGCTTGG; rev: ACAGCAAGGCGAAAAAGGAT,

il17a; for: TGAGCTTCCCAGATCACAGA; rev: TCCAGAAGGCCCTCAGACTA,

foxp3: for: GCGAAAGTGGCAGAGAGGTA; rev: TCCAAGTCTCGTCTGAAGGC,

il6: for: CAAGTCGGAGGCTTAATTACACATG; rev: ATTGCCATTGCACAACTCTTTTCT.

### Statistical Analysis

Data were analyzed for Gaussian distribution using GraphPad 8.0 (GraphPad Software Inc.). Data with Gaussian distribution were assessed for statistical significance using Student’s T-test, data sets with unequal variances were analyzed using Student’s T-test with Welch’s correction, and data without Gaussian distribution were analyzed using Mann-Whitney u-test. Data with more than two groups were analyzed with 1-way ANOVA, data with more than 2 groups and conditions were analyzed using 2-way ANOVA.

## Results

### PTEN^ΔDC^ Mice Show Increased Mortality in DSS-Induced Colitis and Colitis Associated Colon Cancer (CAC)

We recently found that the PI3K/PTEN signaling pathway is driving CD8α^+^DCs expressing PD-L1 and PD-L2 and impairing efficient CD8 T-cell responses in two different cancer models in a myeloid-PTEN (PTEN^Δmye^) specific mouse model (13).

**Figure 1:**
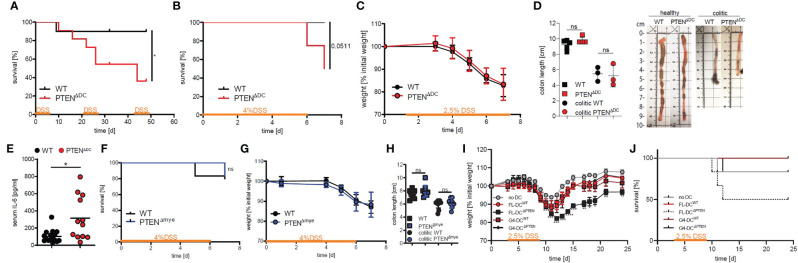
PI3Kinase activation in DCs results in increased mortality in DSS-induced colitis. **(A)** Kaplan-Meier-Estimator of survival during chronic DSS-induced colitis (cycle 1 and 2: 2.5%, cycle 3: 2% DSS; n=10-11 per group), *p<0.05, log-rank test. **(B)** Kaplan-Meier-Estimator of survival during acute DSS-induced colitis (n=6-8 per group), log-rank test. **(C)** weight change during acute DSS-induced colitis (n=4-5 per group). **(D)** colon length on day 7 after start of DSS supplement (colitic) and healthy control animals (n=3-5 per group), ns - not significant, 1-way ANOVA with Bonferroni post-test and representative pictures of colon from healthy or colitic animals **(E)** serum levels of IL-6 measured on day 7 after start of DSS supplementation (n=12-16 per group), *p<0.05, T-test with Welch’s correction. **(F)** Kaplan-Meier-Estimator of survival during acute DSS-induced colitis in PTEN^Δmye^ mice (n=5-6 per group), ns - not significant, log-rank test. **(G)** weight change during acute DSS-induced colitis in PTEN^Δmye^ mice (n=5 per group). **(H)** colon length on day 7 after start of DSS supplement (colitic) and healthy control PTEN^Δmye^ mice (n=8-12 per group), ns - not significant, 2-way ANOVA with Bonferroni post-test. **(I)** weight change during acute DSS-induced colitis after DC transfer (n=6 per group), *p<0.05, **p<0.01, ***p<0.001, ****p<0.0001, 2-way ANOVA with Dunnett’s multiple comparisons. **(J)** survival of mice as shown in **(I)**.

In order to elucidate the role of PI3K activity specifically in DCs in colitis-associated colon cancer (CAC), we made use of a DC-specific cre-deleter strain generating PTEN^ΔDC^ mice. Unlike WTs and PTEN^Δmye^ mice (13), PTEN^ΔDC^ mice succumbed significantly earlier during the course of CAC. Mortality occurred already at the first administration of DSS and was independent of gender (**Figure S1A**). Since at this early time point tumors are not yet formed, we hypothesized that the observed phenotype was caused by an inflammatory response. In acute and chronic models of DSS-induced colitis about half of PTEN^ΔDC^ mice succumbed, whereas cre^-/-^ littermates (hereafter named wildtypes, WT) were mostly unaffected (**Figures 1A, B**). Due to the observed mortality in the acute model when 4% DSS were used (**Figure 1B**), we lowered the percentage to 2.5% DSS in all further experiments involving one cycle of DSS supplementation.

All mice lost around 20% weight by day 7 (**Figure 1C**) and showed the same extent of colon shrinking (**Figure 1D**) and similar histological changes (loss of crypts, hypertrophy of the lamina propria and cell influx; **Figure S1C**). In PTEN^ΔDC^ mice, we found elevated IL-6 serum levels (**Figure 1E**), but no changes in IL-33 or CXCL-1 (also known as KC), which previously have been shown to be increased during colitis (16–20) (**Figure S1B**).

To exclude the possibility that other myeloid PTEN-deficient cells apart from DCs such as macrophages or neutrophils would contribute to the observed phenotype, we made use of PTEN^fl/fl^ LysMCre^+/-^ mice (PTEN^Δmye^), where the deletion more broadly targets myeloid cells, and induced colitis with a DSS concentration of 4%. In contrast to their increased mortality in the AOM/DSS-induced CAC model (13), PTEN^Δmye^ mice did not show increased mortality, during acute (**Figure 1F**) or chronic colitis (after 3x cycles of DSS, data not shown).

PTEN^Δmye^ mice showed a similar weight loss (**Figure 1G**) and colon shortening as WT littermate controls (**Figure 1H**). Furthermore, we could not detect any changes in plasma IL-6 or CXCL1 levels (**Figure S1D**). These data suggest the involvement of a cell type that is fate-mapped by the CD11c-cre but not, or just insignificantly, by the LysMCre as the cause of the observed phenotype in the DSS-induced colitis model.

To narrow down the responsible cell type and to rule out any contribution of potentially targeted T or B cells (6, 21) in the CD11c-cre deleter strain, we generated DCs from bone marrow using either Flt3L (FL-DCs), which is necessary for systemic and intestinal conventional DC (cDC) development (2) or GM-CSF/IL-4 (G4-DCs) and adoptively transferred these into C57BL/6J recipients 3 days prior to colitis induction with DSS. During the course of disease, G4-DC recipients – regardless of donor genotype - lost weight to the same extent as controls that did not receive DCs. In contrast, WT mice that received PTEN^-/-^ FL-DCs lost significantly more weight than untreated controls starting at day 12 (day 9 post DSS supplementation) and did not fully recover (**Figure 1I**). In addition, these recipients had an increased mortality rate similar to that observed in PTEN^ΔDC^ mice during the course of colitis (**Figures 1A, J**), suggesting that Flt3L-dependent PTEN-deficient dendritic cells are sufficient to transfer the observed phenotype.

Furthermore, we did not detect any difference in weight loss between mice that did receive DCs (FL-DC^WT or ΔPTEN^ or G4-DCs^WT or ΔPTEN^) or mice that did not receive DCs until day 10 (day 7 post start of DSS, **Figure S1E**). There was also no significant difference in serum IL-6 or colon length between the treated groups (**Figures S1F, G**). We were not able to measure IL-33 in the serum of these animals.

Taken together, PTEN^ΔDC^ mice show increased mortality and plasma IL-6 levels during colitis without any impact on weight loss or histological changes compared to WTs. The observed phenotype was furthermore independent of any contribution from PTEN-deficient macrophages or neutrophils. Importantly, Flt3L-dependent DCs but not GM-CSF/IL-4 DCs are sufficient to transfer the survival phenotype.

### DC-PI3K Drives Th1 Responses and IFNγ Production in Acute DSS-Induced Colitis

To rule out that the intestinal barrier function was compromised in PTEN^ΔDC^ mice before the induction of colitis, we assessed the barrier function by measuring the transfer of FITC-dextran into the blood after oral gavage (14). We did not observe any differences in FITC-dextran that was transferred from the intestinal lumen into the blood in PTEN^ΔDC^ mice compared to their cre^-/-^ WT littermates (**Figure S2A**). In addition, we performed colonoscopy four days after withdrawal of DSS and found similar granularity, increased plasticity, fibrin deposition and loss of visible vascularity in both genotypes (**Figure S2B**).

**Figure 2:**
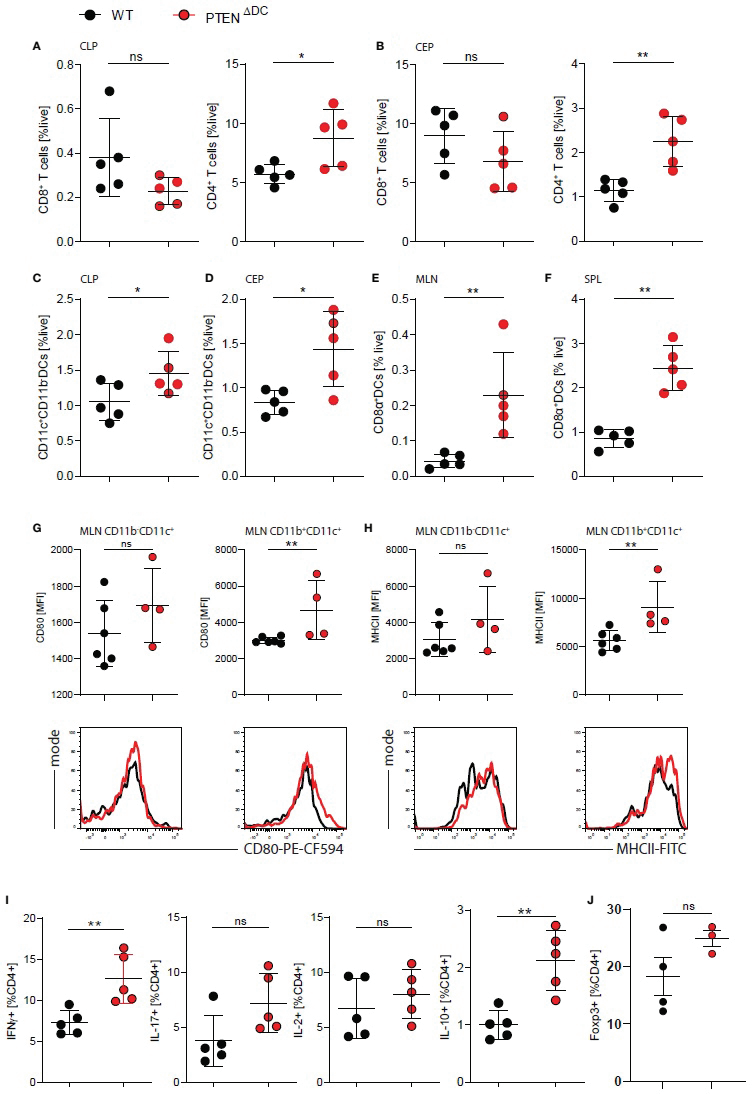
DC-PI3K drives CD4^+^ T cell responses in the inflamed colon. **(A)** CD8^+^ and CD4^+^ T cells in colon lamina propria (CLP) and **(B)** colon epithelium (CEP) on day 7 of DSS-induced colitis. **(C)** DCs in CLP, **(D)** CEP, **(E)** mesenteric lymph nodes (MLN), **(F)** spleen of colitic PTEN^ΔDC^ mice. **(A–F)** n=5 per group), *p<0.05, **p<0.01, ns - not significant, Mann-Whitney test. **(G)** surface expression and representative histograms of CD80 and **(H)** MHC-II (I-A/I-E^b^) on MLN CD11b^+/-^CD11c^+^ DCs expressed as MFI, **(G–H)** n=4-6 per group), **p<0.01, ns - not significant, Mann-Whitney test. **(I)** Quantification of intracellular cytokines in PMA/ionomycin restimulated CD4^+^ T cells from MLN of colitic mice, (n=5 per group) **p<0.01, ns - not significant, Mann-Whitney test. **(J)** Quantification of intracellular Foxp3 staining in CD4^+^ T cells from MLN of colitic mice, (n=3-4 per group) ns - not significant, Mann-Whitney test.

Next, we analyzed the abundance of CD4^+^ and CD8^+^ T cells in the colon and found increased percentages of CD4^+^, but not CD8^+^ T cells in the lamina propria among all live cells (CLP, **Figure 2A**) as well as colon epithelium (CEP, **Figure 2B**) of PTEN^ΔDC^ mice. Systemically, DC-PI3K did not affect CD4^+^ T cells numbers in the spleens (SPL) or mesenteric lymph nodes (MLN) of PTEN^ΔDC^ mice (**Figures S2C, D**). We did observe a reduction of CD8^+^ T cells numbers in the secondary lymphoid organs during DSS-induced colitis but not in healthy animals (**Figures S2C, D** and data not shown). Regardless of disease status, conventional (CD8α^+^)CD11c^+^DCs, which is the main DC subset increased by enhanced PI3K signaling (6), were increased in spleens, mesenteric lymph nodes, colon lamina propria and epithelium (**Figures 2C**–**F**).

Additionally, we analyzed the antigen presenting and co-stimulatory capacity of these DCs in the secondary lymphoid organs and found increased surface expression of CD80 and MHCII on CD11c^+^(CD11b^+^) lymph node DCs (**Figure 2G, H**) and increased CD80 expression on CD11c^+^ DCs in the spleens of colitic PTEN^ΔDC^ mice (**Figures S2E, F**). This coincided with significantly increased mRNA levels of *il10*, but not *ifng*, *il17* or *foxp3* in the CLP of PTEN^ΔDC^ mice (**Figure S2G**), suggesting an increased CD4^+^ T cell response upon PI3K activation in DCs, but also a compensatory activation of regulatory T cells (Tregs).

We next assessed T cell responses in colitic mice and found increased production of IFNγ after CD3/CD28 *ex vivo* restimulation of splenocytes from PTEN^ΔDC^ mice (**Figure S2H**). In addition, we observed an increase in IFNγ release when we co-cultured CD11c^+^ cells from PTEN^ΔDC^ mice with CD4^+^ OTII T cells in the presence of ovalbumin (OVA) and LPS (**Figure S2I**), showing that activation of PI3K signaling in DCs drives IFNγ production by CD4^+^ T cells.

Furthermore, we found higher percentage of IFNγ^+^ and IL10^+^/Foxp3^+^ CD4^+^ T cells in MLN (**Figures 2I, J**) and spleen (**Figure S2J**) of colitic PTEN^ΔDC^ mice, without any differences in percentage of IL2^+^ or IL-17^+^ CD4^+^ T cells. These data suggest that PI3K signaling in DCs drives co-stimulatory signals, thereby enhancing Th1 and compensatory T_reg_ responses and resulting in increased mortality during gut inflammation.

To test whether increased activation and expression of costimulatory ligands upon stimulation of DCs was PI3K dependent, we stimulated FL-DCs with various TLR ligands *in vitro* but observed no changes in neither surface expression of CD40, CD80, CD86 and MHCII nor IL-6 production by PTEN^-/-^ DCs (data not shown). Taken together, these results suggest an increased ability of PTEN^-/-^ DCs to express costimulatory signals during colitis, thereby driving Th1 responses and CD4^+^ T cell infiltration into the gut.

### Antibiotic Treatment or IL-6R Blockade Abrogates Mortality in PTEN^ΔDC^ Mice During Colitis

**Figure 3:**
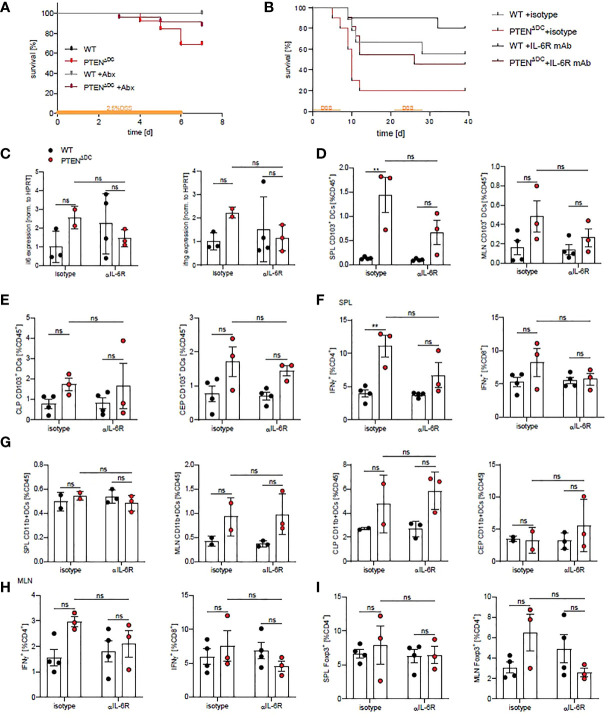
Mortality in PTEN^ΔDC^ mice is rescued by blocking of IL-6R signaling. **(A)** Kaplan-Meier-Estimator of survival during acute DSS-induced colitis during antibiotics treatment (n=13-27). **(B)** Kaplan-Meier-Estimator of survival during chronic DSS-induced colitis treated with αIL-6R (clone 15A7) or isotype (n=9-11 per group). **(C)** Expression of indicated genes in CLP of treated PTEN^ΔDC^ mice during colitis as shown in **(B)** (n=2-4), **(D)** CD103^+^DCs in spleen (SPL) and mesenteric LN (MLN) and **(E)** in CLP and CEP of colitic mice treated with αIL-6R (clone 15A7, BioXCell) or isotype control (day 6). **(F)** Splenic IFNγ^+^CD4^+^ and IFNγ^+^CD8^+^ T cells. **(G)** CD11b^+^DCs in spleen (SPL), mesenteric LN (MLN), CLP and CEP of colitic mice treated with αIL-6R shown in **(B)**. **(H)** MLN IFNγ^+^CD4^+^ and IFNγ^+^CD8^+^ T cells. **(I)** Foxp3^+^CD4^+^ T cells in spleens and MLN. (**C**–**I** n=3-4 per group) **p<0.01, ns - not significant, 2-way ANOVA with Tukey’s multiple comparison.

Aberrant innate immune responses to the microbial flora and the quality and quantity of the intestinal microbiota severely influence the development and course of intestinal inflammation (22–25), which has been shown to be promoted by MyD88-dependent IL-6 production in innate immune cells. Further, IL-6 is increased in the majority of IBD patients and correlates with disease severity (3, 26). Since we found increased expression of costimulatory molecules and IL-6 levels in colitic PTEN^ΔDC^ mice, we asked whether ablation of the microbiota or of IL-6R signaling rescues the high mortality observed in PTEN^ΔDC^ mice during colitis. Treatment of PTEN^ΔDC^ mice with antibiotics (Abx) reduced the observed mortality during DSS-colitis, whereas WT mice remained unaffected (**Figure 3A**).

IL-6 signaling by DCs has been shown to play a major role in inducing microbiota-directed T cell responses (26) and has been shown to be required for Th1 responses during intestinal inflammation (27–30). Therefore, we treated PTEN^ΔDC^ and WT mice with an IL-6R blocking antibody twice a week during 2 cycles of DSS/water supplementation, to block all 3 ways of IL-6/IL-6R signaling [classical signaling, trans-signaling and trans-presentation, reviewed in (31)]. Mortality during the course of colitis with IL-6R blockade was substantially decreased in both WT and PTEN^ΔDC^ mice. Notably, PTEN^ΔDC^ mice treated with blocking IL-6R antibody showed similar mortality to DSS-induces colitis as WT mice without the blocking antibody (**Figure 3B**). In addition, transcription of *il6* and *ifng* was slightly reduced in PTEN^ΔDC^ mice treated with IL-6R blocking antibodies compared to isotype treated controls (**Figure 3C**) and there was a tendency of IL-6R blockade in reducing CD103^+^DCs in spleens and in MLN (**Figure 3D**), but not in colon lamina propria and epithelium (**Figure 3E**). The percentage of CD11b^+^DCs was not affected in any of the analyzed organs (**Figure 3G**). These results suggest that sensing of the microbiota and enhanced IL-6 production cause the observed mortality in PTEN^ΔDC^ mice during DSS-induced colitis, independent of gut barrier function (**Figure S2B**).

In addition, we analyzed T cell responses after IL-6R blockade at an early time point when all animals were still alive and found a tendency of IL-6R blockade to decrease IFNγ producing T cells in spleens and MLN of PTEN^ΔDC^ compared to WT mice at day 6 (**Figures 3F, H**). Foxp3 expressing T_regs_ were slightly increased in MLN of PTEN^ΔDC^ mice and were decreased upon IL-6R blockade (**Figure 3I**).

## Discussion

DCs constitute an important cellular part of the immune system and are necessary to maintain immune homeostasis, especially at sites of exposure to commensals like the lung, the skin or the gut. During infection and inflammation DCs may also contribute to prolongation of detrimental responses resulting in chronic inflammation. Therefore, understanding the regulation of DC function in homeostasis and pathology is necessary in order to be able to manipulate DC responses. The PI3K signaling pathway has been shown to regulate the development as well as the immunologic capacity of many types of myeloid cells, including DCs.

The present study aimed at investigating the regulation of DC function by the PI3K/PTEN pathway during acute and chronic intestinal inflammation. Therefore, we deleted PTEN in DCs, which results in unopposed PI3K signaling in these cells and asked whether this would affect induction and progression of disease. In contrast to the anti-inflammatory, beneficial immunophenotype observed in PTEN-deficient macrophages (7, 10–12) in infection and inflammation models, we found a high incidence of mortality during DSS-induced colitis and CAC. This is in contrast to our previous work, where we could show that myeloid PTEN deficiency drives immunosuppressive DCs in CAC (13). Our data also show that myeloid PTEN-deficiency does not protect from DSS-induced colitis or affect survival. Most likely, DCs that are only marginally targeted by the LysMCre, are responsible for the observed phenotype in PTEN^ΔDC^ mice.

Mortality during colitis and CAC started around day 6 in PTEN^ΔDC^ mice suggesting an adaptive rather than an immediate innate immune response, as for example caused by neutrophils or monocytes. Morphological changes in the gut as well as gut barrier function were similar between the genotypes excluding the possibility of detrimental preexisting defects in the large intestine of PTEN^ΔDC^ mice.

The cell type responsible for the observed mortality is likely to be Flt3L-dependet dendritic cells, since adoptive transfer of PTEN^-/-^ FL-DCs, but not GM-CSF/IL-4 derived DCs, was sufficient to induce mortality and weight loss after colitis induction in WT recipients. In contrast to a previously published report (4), we did not observe a worsening of disease after transfer of WT GM-CSF/IL-4 DCs.

Colitic PTEN^ΔDC^ mice showed increased plasma levels of IL-6, which we did not observe in myeloid PTEN-deficient mice. Furthermore, CD8α^+^ and CD103^+^DCs were increased in number in secondary lymphoid organs as well as in the colon lamina propria and epithelium in healthy and colitic mice. This is in accordance with previous findings showing that downstream of Flt3/CD135, the receptor for Flt3L, PI3K/AKT/mTOR signaling drives CD8α^+^ and CD103^+^DC development (6).

Furthermore, we found increased numbers of CD4^+^ T cells in the colons of PTEN^ΔDC^ mice and elevated levels of IL-10 transcripts in the lamina propria of these mice. In the secondary lymphoid organs we observed less CD8^+^ T cells in colitic but not healthy PTEN^ΔDC^ mice. In addition, CD4^+^ T cells from colitic PTEN^ΔDC^ mice produced more IL-10 and IFNγ, but not IL-2 or IL-17a after *ex vivo* PMA/Ionomycin re-stimulation. This coincided with elevated expression of CD80 and MHCII on splenic CD11c^+^(CD11b^-^) and MLN CD11c^+^(CD11b^+^) cells suggesting an increased capacity of PTEN^-/-^ DCs to present antigen and co-stimulate CD4^+^ T cells thus inducing an increased IL-10 and IFNγ response in these cells.

Flt3L-driven bone marrow-derived dendritic cells (FL-DCs) generated from PTEN^ΔDC^ mice however did not express higher levels of CD40, MHCII, CD80 and CD86 after *in vitro* stimulation with different TLR ligands. Further experiments are required to elucidate the mechanism as to how these activation markers are elevated on splenic and MLN DCs of PTEN^ΔDC^ mice. Previous findings have shown a protective effect of adoptively transferred, TLR9-stimulated DCs into DSS-treated mice (32). In our transfer experiments we used untreated cells and could not observe additional protection upon (untreated) WT FL-DC transfer compared to controls that did not receive DCs.

DSS colitis has been shown to depend on the presence of the microbiota, since mice treated with antibiotics (abx) or germ-free mice do not develop colitis after DSS supplementation (20). Furthermore, microbiota dependent TLR-mediated activation and IL-6 production by DCs leading to T cell activation are necessary for colitis induction (26). In order to assess whether PI3K signaling after microbiota-mediated DC activation during DSS colitis results in enhanced mortality, we treated mice with antibiotics during DSS-induced colitis and could increase the survival of PTEN^ΔDC^ mice to the level of WT mice. In addition, treatment with an IL-6R blocking antibody was able to rescue the observed mortality in PTEN^ΔDC^ mice.

IL-6 is a pleiotropic cytokine with pro-inflammatory as well as regenerating features (3, 33). IL-6 is induced during gut injury (5, 33) and is necessary for intestinal repair after wounding which might also give rise to intestinal tumor formation (34, 35). In addition, IL-6R signaling in T cells is a prerequisite for induction of T cell mediated colitis since its blockade has been shown to abrogate proliferation of CD4^+^ or CD8^+^ T cells, IFNγ production and induction of colitis in models of T cell transfer colitis (27–29, 36, 37). Mechanistically, IL-6 renders T cells insensitive to Treg mediated inhibition (28). Blockade of IL-6R signaling in PTEN^ΔDC^ mice tended to reduce IFNγ production by CD4^+^ and CD8^+^ T cells in both mesenteric lymph nodes and the spleen, without affecting splenic T_reg_ numbers in PTEN^ΔDC^ mice. IFNγ levels in T cells from control PTEN^ΔDC^ mice were up to 2-fold elevated and IFNγ KO mice are protected from colitis induction (38), highlighting the pivotal role of Th1 mediated responses in the pathogenesis of colitis, which is to some extent driven by PI3K signaling in dendritic cells. Nevertheless, we cannot rule out any effects of IL-6 on additional cell types such as intestinal epithelial cells or innate lymphoid cells (33, 39), since IL-6 is a cytokine which exerts pleiotropic effects including the regulation of intestinal barrier function (40, 41). We did not observe a change in barrier function with respect to FITC-labeled dextran, but this might be limited to the size and nature of dextrans compared to bacteria or other pathogens.

In addition, in a recent study Czarnewski and colleagues (42) using the DSS colitis model identified key genes by RNA sequencing of colonic tissue allowing them to stratify patients suffering from ulcerative colitis into two distinct groups which differed in their response to commonly used biological therapies. IL-6 was found among the genes highly expressed in patients not responding to either TNFα or integrin α4β7 blockade. These data support the notion of potential beneficial effects of blocking additional inflammatory mediators, like IL-6, in these patients.

Taken together, we identified the PI3K/PTEN signaling pathway in dendritic cells as a driver of IL-6 mediated Th1 responses which is triggered upon microbiota dependent inflammation in a model of DSS-induced colitis.

## Data Availability Statement

The raw data supporting the conclusions of this article will be made available by the authors, without undue reservation.

## Ethics Statement

The animal study was reviewed and approved by the Austrian Federal Ministry for Education, Science and Research.

## Author Contributions

MKut and GS conceived and designed the study. DH performed all necessary experiments for the revision, generated and analysed the data and revised the manuscript. MKut, CY, JSB, AV, DH, HP, SP, MW, HD, LM, KS and AH performed in vivo and in vitro experiments. MKut, JSB, AV, DH, KS, AH performed and analyzed flow cytometry data. CY, MW, SP, HP performed and analyzed gene expression data. DH, MH, MKer, MKie, HD, LM, OS and MS contributed to experimental design and data interpretation. CY, HP, SP and MW helped with mouse colony management. MS performed and quantified blood counts. ML performed colonoscopies and scored the animals. OS provided conceptual input for experimental design and data interpretation. MKut, DH and GS wrote the manuscript and all authors read, revised and approved the manuscript.

## Funding

The present study was funded by the Austrian Science Fund (FWF) projects P24802, P30026 to GS and P31106 to MKu.

## Conflict of Interest

The authors declare that the research was conducted in the absence of any commercial or financial relationships that could be construed as a potential conflict of interest.

## Publisher’s Note

All claims expressed in this article are solely those of the authors and do not necessarily represent those of their affiliated organizations, or those of the publisher, the editors and the reviewers. Any product that may be evaluated in this article, or claim that may be made by its manufacturer, is not guaranteed or endorsed by the publisher.

## Supplementary Material

Supplementary Figure 1
**(A)** Kaplan-Meier-Estimator of survival during AOM/DSS-induced colorectal cancer in male (n=19 per group) and female PTEN^ΔDC^ mice, (n=8-11 per group) *p<0.05, **p<0.01, log-rank test. **(B)** serum levels of KC/CXCL1 and IL-33 in PTEN^ΔDC^ mice measured on day 7 after start of DSS supplementation (n=10-17), ns - not significant, Mann-Whitney test. **(C)** H&E stainings of FFPE sections of colon swiss rolls from colitic mice on day 7 and day 48, black bars indicate 500µm. **(D)** serum levels of IL-6 (n=5-6) and KC/CXCL1 (n=6-9) in PTEN^Δmye^ measured on day 7 after start of DSS supplementation, ns - not significant, Mann-Whitney test. **(E)** weight change, **(F)** serum levels of IL-6, and **(G)** colon length during acute DSS-induced colitis after DC transfer until day 10 (n=6 per grp.

Supplementary Figure 2
**(A)** quantification of gut barrier function measured by the transfer for orally gavaged FITC-dextran into the serum of healthy mice (n=20-21), ns - not significant, Mann-Whitney test. **(B)** representative images taken during colonoscopy on day 10 after start of DSS supplement and quantification of inflammation score in healthy and colitic mice, AU-arbitrary units. **(C)** CD8^+^ and CD4^+^ T cells in spleens and **(D)** MLN of colitic PTEN^ΔDC^ mice on day 7 of DSS-colitis, (n=5 per group) **p<0.01, ns - not significant, Mann-Whitney test. **(E)** surface expression and representative histograms of CD80 and **(F)** MHC-II (I-A/I-E^b^) on splenic CD11b^+/-^CD11c^+^ DCs expressed as MFI, (n=4-6 per group) **p<0.01, ns - not significant, Mann-Whitney test. **(G)** normalized gene expression in whole colon lamina propria (CLP) cells for indicated genes, (n=5 per group) **p<0.01, ns - not significant, Mann-Whitney test. **(H)** IFNγ levels measured in supernatants of anti-CD3/CD28 restimulated splenocytes from colitic PTEN^ΔDC^ mice, (n=3-6 per group) ***p<0.001, ns- not significant, 2-way ANOVA with Bonferroni’s multiple comparison, **(I)** IFNγ levels measured in supernatants of splenic CD11c^+^ DCs co-cultured with CD4^+^OT-II T cells in the presence of LPS and ovalbumin, (n=4-5 per group) *p<0.05, Mann-Whitney test. **(J)** Quantification of intracellular cytokines in PMA/ionomycin restimulated CD4^+^ T cells in spleens of colitic PTEN^ΔDC^ mice, as well Foxp3^+^CD4^+^ T cells (n=4-5 per group) ** p<0.01, ns - not significant, Mann-Whitney test. **(K)** gating to identify cells in **Figures 2A**–**F**, **Figures S2C, D**. **(L)** gating to identify cells in **Figures 2G, H** , **Figures S2E, F**. **(M)** gating to identify cells in **Figure 2I**, **Figure S2H**, numbers in blots indicate percentage of gated cells from the parental gate.

Supplementary Figure 3
**(A)** gating to identify cells in **Figures 3B, C**, **(B)** gating to identify cells in **Figures 3D, E**.

## Abbreviations

PI3K, Phosphatidylinositol (3,4,5) trisphosphate kinase; PTEN, phosphatase and tensin homolog deleted on chromosome 10; CAC, colitis-associated colon cancer; AOM, azoxymethane; DSS, dextran sodium sulphate; DC, dendritic cell; OVA, ovalbumin; APC, antigen-presenting cell; SPL, spleen; MLN, mesenteric lymph node; CEP, colon epithelium; CLP, colon lamina propria.

## References

[1] Maloy KJ Powrie F. Intestinal Homeostasis and its Breakdown in Inflammatory Bowel Disease. Nature (2011) 474:298–306. doi: 10.1038/nature10208 21677746

[2] Stagg AJ. Intestinal Dendritic Cells in Health and Gut Inflammation. Front Immunol (2018) 9:2883. doi: 10.3389/fimmu.2018.02883 30574151 PMC6291504

[3] Waldner MJ Neurath MF. Master Regulator of Intestinal Disease: IL-6 in Chronic Inflammation and Cancer Development. Semin Immunol (2014) 1:75–9. doi: 10.1016/j.smim.2013.12.003 24447345

[4] Berndt BE Zhang M Chen G-H Huffnagle GB Kao JY. The Role of Dendritic Cells in the Development of Acute Dextran Sulfate Sodium Colitis. J Immunol (2007) 179:6255–62. doi: 10.4049/jimmunol.179.9.6255 17947701

[5] Qualls JE Tuna H Kaplan AM Cohen DA. Suppression of Experimental Colitis in Mice by CD11c+ Dendritic Cells. Inflammation Bowel Dis (2009) 15:236–47. doi: 10.1002/ibd.20733 18839426

[6] Sathaliyawala T O’Gorman WE Greter M Bogunovic M Konjufca V Hou ZE. Mammalian Target of Rapamycin Controls Dendritic Cell Development Downstream of Flt3 Ligand Signaling. Immunity (2010) 33:597–606. doi: 10.1016/j.immuni.2010.09.012 20933441 PMC2966531

[7] Luyendyk JP Schabbauer GA Tencati M Holscher T Pawlinski R Mackman N. Genetic Analysis of the Role of the PI3K-Akt Pathway in Lipopolysaccharide-Induced Cytokine and Tissue Factor Gene Expression in Monocytes/Macrophages. J Immunol (2008) 180:4218–26. doi: 10.4049/jimmunol.180.6.4218 PMC2834303 18322234

[8] Schabbauer G Matt U Günzl P Warszawska J Furtner T Hainzl E. Myeloid PTEN Promotes Inflammation But Impairs Bactericidal Activities During Murine Pneumococcal Pneumonia. J Immunol (2010) 185:468–76. doi: 10.4049/jimmunol.0902221 20505137

[9] Weichhart T Costantino G Poglitsch M Rosner M Zeyda M Stuhlmeier KM. The TSC-mTOR Signaling Pathway Regulates the Innate Inflammatory Response. Immunity (2008) 29:565–77. doi: 10.1016/j.immuni.2008.08.012 18848473

[10] Sahin E Haubenwallner S Kuttke M Kollmann I Halfmann A Dohnal AM. Correction: Macrophage PTEN Regulates Expression and Secretion of Arginase I Modulating Innate and Adaptive Immune Responses. J Immunol (2014) 193:5350–0. doi: 10.4049/jimmunol.1490039 PMC4120896 25015834

[11] Sahin E Brunner JS Kral JB Kuttke M Hanzl L Datler H. Loss of Phosphatase and Tensin Homolog in APCs Impedes Th17-Mediated Autoimmune Encephalomyelitis. J Immunol (2015) 195:2560–70. doi: 10.4049/jimmunol.1402511 26246144

[12] Kral JB Kuttke M Schrottmaier WC Birnecker B Warszawska J Wernig C. Sustained PI3K Activation Exacerbates BLM-Induced Lung Fibrosis *via* Activation of Pro-Inflammatory and Pro-Fibrotic Pathways. Sci Rep (2016) 6:1–16. doi: 10.1038/srep23034 26971883 PMC4789787

[13] Kuttke M Sahin E Pisoni J Percig S Vogel A Kraemmer D. Myeloid PTEN Deficiency Impairs Tumor-Immune Surveillance *via* Immune-Checkpoint Inhibition. Oncoimmunology (2016) 5:1–13. doi: 10.1080/2162402X.2016.1164918 PMC5006931 27622019

[14] Gupta JN RA. Analysis of Intestinal Permeability in Mice. bioprotocols (2014) 4:219–32. doi: 10.21769/BioProtoc.1289

[15] Ruijter JM Ramakers C Hoogaars WMH Karlen Y Bakker O van den hoff MJB. Amplification Efficiency: Linking Baseline and Bias in the Analysis of Quantitative PCR Data. Nucleic Acids Res (2009) 37:15–12. doi: 10.1093/nar/gkp045 19237396 PMC2665230

[16] Pastorelli L Garg RR Hoang SB Spina L Mattioli B Scarpa M. Epithelial-Derived IL-33 and its Receptor ST2 Are Dysregulated in Ulcerative Colitis and in Experimental Th1/Th2 Driven Enteritis. Proc Natl Acad Sci USA (2010) 107:8017–22. doi: 10.1073/pnas.0912678107 PMC2867895 20385815

[17] Pastorelli L De Salvo C Cominelli MA Pizarro TT Vecchi M. Novel Cytokine Signaling Pathways in Inflammatory Bowel Disease: Insight Into the Dichotomous Functions of IL-33 During Chronic Intestinal Inflammation. Therap Adv Gastroenterol (2011) 4:311–23. doi: 10.1177/1756283X11410770 PMC3165208 21922030

[18] Liew FY Pitman NI McInnes IB. Disease-Associated Functions of IL-33: The New Kid in the IL-1 Family. Nat Rev Immunol (2010) 10:103–10. doi: 10.1038/nri2692 20081870

[19] Shea-Donohue T Thomas K Cody MJ Zhao A Detolla LJ Kopydlowski KM. Mice Deficient in the CXCR2 Ligand, CXCL1 (KC/GRO-α), Exhibit Increased Susceptibility to Dextran Sodium Sulfate (DSS)-Induced Colitis. Innate Immun (2008) 14:117–24. doi: 10.1177/1753425908088724 PMC2614619 18713728

[20] Chassaing B Aitken JD Malleshappa M Vijay-Kumar M. Dextran Sulfate Sodium (DSS)-Induced Colitis in Mice. Curr Protoc Immunol (2014) 104:15–25. doi: 10.1002/0471142735.im1525s104 PMC3980572 24510619

[21] Golinski ML Demeules M Derambure C Riou G Maho-Vaillant M Boyer O. CD11c+ B Cells Are Mainly Memory Cells, Precursors of Antibody Secreting Cells in Healthy Donors. Front Immunol (2020) 11:32. doi: 10.3389/fimmu.2020.00032 32158442 PMC7051942

[22] Kitajima S Morimoto M Sagara E Shimizu C Ikeda Y. Dextran Sodium Sulfate-Induced Colitis in Germ-Free IQI/Jic Mice. Exp Anim (2001) 50:387–95. doi: 10.1538/expanim.50.387 11769541

[23] Håkansson Å Tormo-Badia N Baridi A Xu J Molin G Hagslätt ML. Immunological Alteration and Changes of Gut Microbiota After Dextran Sulfate Sodium (DSS) Administration in Mice. Clin Exp Med (2014) 15:107–20. doi: 10.1007/s10238-013-0270-5 PMC4308640 24414342

[24] Hernández-Chirlaque C Aranda CJ Ocón B Capitán-Cañadas F Ortega-González M Carrero JJ. Germ-Free and Antibiotic-Treated Mice are Highly Susceptible to Epithelial Injury in DSS Colitis. J Crohn’s Colitis (2016) 10:1324–35. doi: 10.1093/ecco-jcc/jjw096 27117829

[25] Roy U Gá Lvez EJC Iljazovic A Huber S Flavell RA Correspondence S. Distinct Microbial Communities Trigger Colitis Development Upon Intestinal Barrier Damage *via* Innate or Adaptive Immune Cells. Cell Rep (2017) 21:994–1008. doi: 10.1016/j.celrep.2017.09.097 29069606 PMC5668567

[26] Feng T Wang L Schoeb TR Elson CO Cong Y. Microbiota Innate Stimulation Is a Prerequisite for T Cell Spontaneous Proliferation and Induction of Experimental Colitis. J Exp Med (2010) 207:1321–32. doi: 10.1084/jem.20092253 PMC2882839 20498021

[27] Yamamoto M Yoshizaki K Kishimoto T Ito H. IL-6 is Required for the Development of Th1 Cell-Mediated Murine Colitis. J Immunol (2000) 164:4878–82. doi: 10.4049/jimmunol.164.9.4878 10779797

[28] Nish SA Schenten D Wunderlich T Pope SD Gao Y Hoshi N. T Cell-Intrinsic Role of IL-6 Signaling in Primary and Memory Responses. Elife (2014) 2014:1–21. doi: 10.7554/eLife.01949 PMC4046568 24842874

[29] Li B Jones LL Geiger TL. IL-6 Promotes T Cell Proliferation and Expansion Under Inflammatory Conditions in Association With Low-Level Rorγt Expression. J Immunol (2018) 201:2934–46. doi: 10.4049/jimmunol.1800016 PMC6324200 30315140

[30] Knop L Frommer C Stoycheva D Deiser K Kalinke U Blankenstein T. Interferon-γ Receptor Signaling in Dendritic Cells Restrains Spontaneous Proliferation of CD4+ T Cells in Chronic Lymphopenic Mice. Front Immunol (2019) 10:140. doi: 10.3389/fimmu.2019.00140 30792713 PMC6374634

[31] Kang S Tanaka T Narazaki M Kishimoto T. Targeting Interleukin-6 Signaling in Clinic. Immunity (2019) 50:1007–23. doi: 10.1016/j.immuni.2019.03.026 30995492

[32] Abe K Nguyen KP Fine SD Mo JH Shen C Shenouda S. Conventional Dendritic Cells Regulate the Outcome of Colonic Inflammation Independently of T Cells. Proc Natl Acad Sci USA (2007) 104:17022–7. doi: 10.1073/pnas.0708469104 PMC2040469 17942668

[33] Kuhn KA Manieri NA Liu TC Stappenbeck TS. IL-6 Stimulates Intestinal Epithelial Proliferation and Repair After Injury. PloS One (2014) 9:1–18. doi: 10.1371/journal.pone.0114195 PMC4257684 25478789

[34] Terzić J Grivennikov S Karin E Karin M. Inflammation and Colon Cancer. Gastroenterology (2010) 138:2101–14. doi: 10.1053/j.gastro.2010.01.058 20420949

[35] Greten FR Grivennikov SI. Inflammation and Cancer: Triggers, Mechanisms, and Consequences. Immunity (2019) 51:27–41. doi: 10.1016/j.immuni.2019.06.025 31315034 PMC6831096

[36] Noguchi D Wakita D Tajima M Ashino S Iwakura Y Zhang Y. Blocking of IL-6 Signaling Pathway Prevents CD4+ T Cell-Mediated Colitis in a Th17-Independent Manner. Int Immunol (2007) 19:1431–40. doi: 10.1093/intimm/dxm114 17981790

[37] Tajima M Wakita D Noguchi D Chamoto K Yue Z Fugo K. IL-6-Dependent Spontaneous Proliferation Is Required for the Induction of Colitogenic IL-17-Producing CD8+ T Cells. J Exp Med (2008) 205:1019–27. doi: 10.1084/jem.20071133 PMC2373835 18426983

[38] Ito R Shin-Ya M Kishida T Urano A Takada R Sakagami J. Interferon-Gamma Is Causatively Involved in Experimental Inflammatory Bowel Disease in Mice. Clin Exp Immunol (2006) 146:330–8. doi: 10.1111/j.1365-2249.2006.03214.x PMC1942055 17034586

[39] Powell N Lo JW Biancheri P Vossenkämper A Pantazi E Walker AW. Interleukin 6 Increases Production of Cytokines by Colonic Innate Lymphoid Cells in Mice and Patients With Chronic Intestinal Inflammation. Gastroenterology (2015) 149:456–67.e15. doi: 10.1053/j.gastro.2015.04.017 25917784 PMC4539618

[40] Grivennikov S Karin E Terzic J Mucida D Yu GY Vallabhapurapu S. IL-6 and Stat3 Are Required for Survival of Intestinal Epithelial Cells and Development of Colitis-Associated Cancer. Cancer Cell (2009) 15:103–13. doi: 10.1016/j.ccr.2009.01.001 PMC2667107 19185845

[41] Guo Y Wang B Wang T Gao L Yang ZJ Wang FF. Biological Characteristics of Il-6 and Related Intestinal Diseases. Int J Biol Sci (2020) 17:204–19. doi: 10.7150/ijbs.51362 PMC7757046 33390844

[42] Czarnewski P Parigi SM Sorini C Diaz OE Das S Gagliani N. Conserved Transcriptomic Profile Between Mouse and Human Colitis Allows Unsupervised Patient Stratification. Nat Commun (2019) 10:1–11. doi: 10.1038/s41467-019-10769-x 31253778 PMC6598981

