# Supplementary material for: PI3K Signaling in Dendritic Cells Aggravates DSS-Induced Colitis
Source: Front Immunol. 2022 Apr 19;13:695576. doi: 10.3389/fimmu.2022.695576 (PMC9063450; doi:10.3389/fimmu.2022.695576)

Supplementary Figure 1

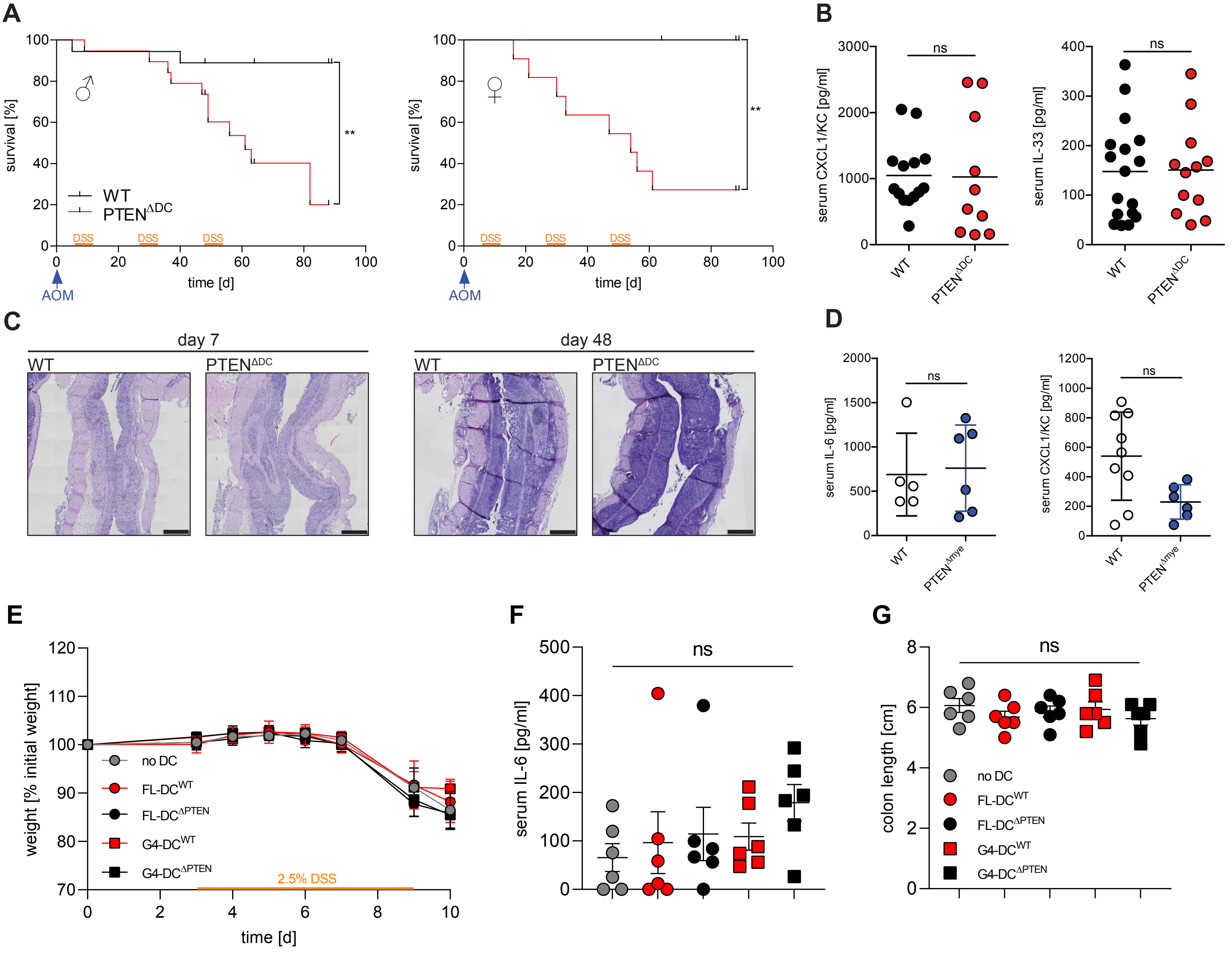

Supplementary Figure 2.

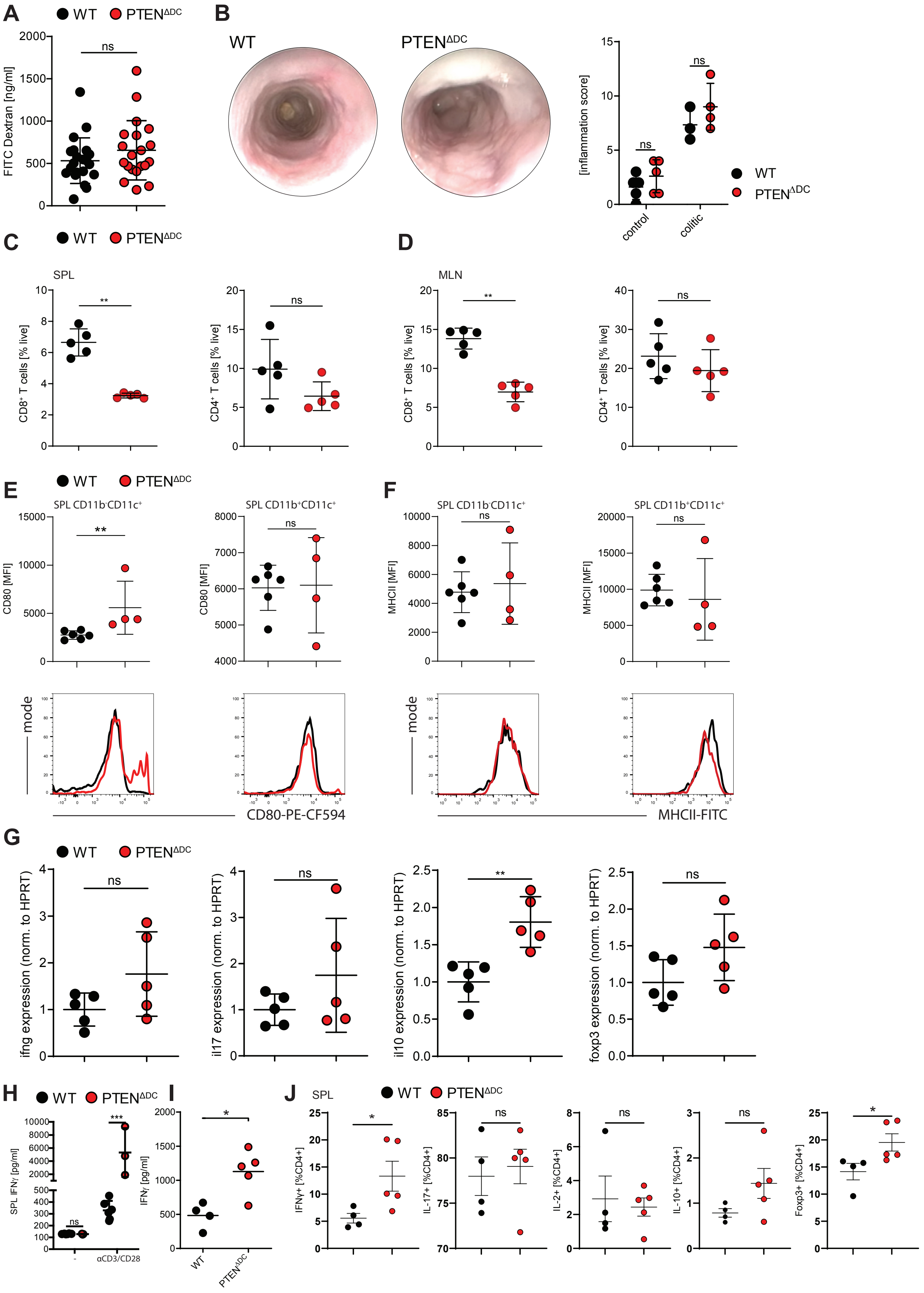

Supplementary Figure 2 continued.

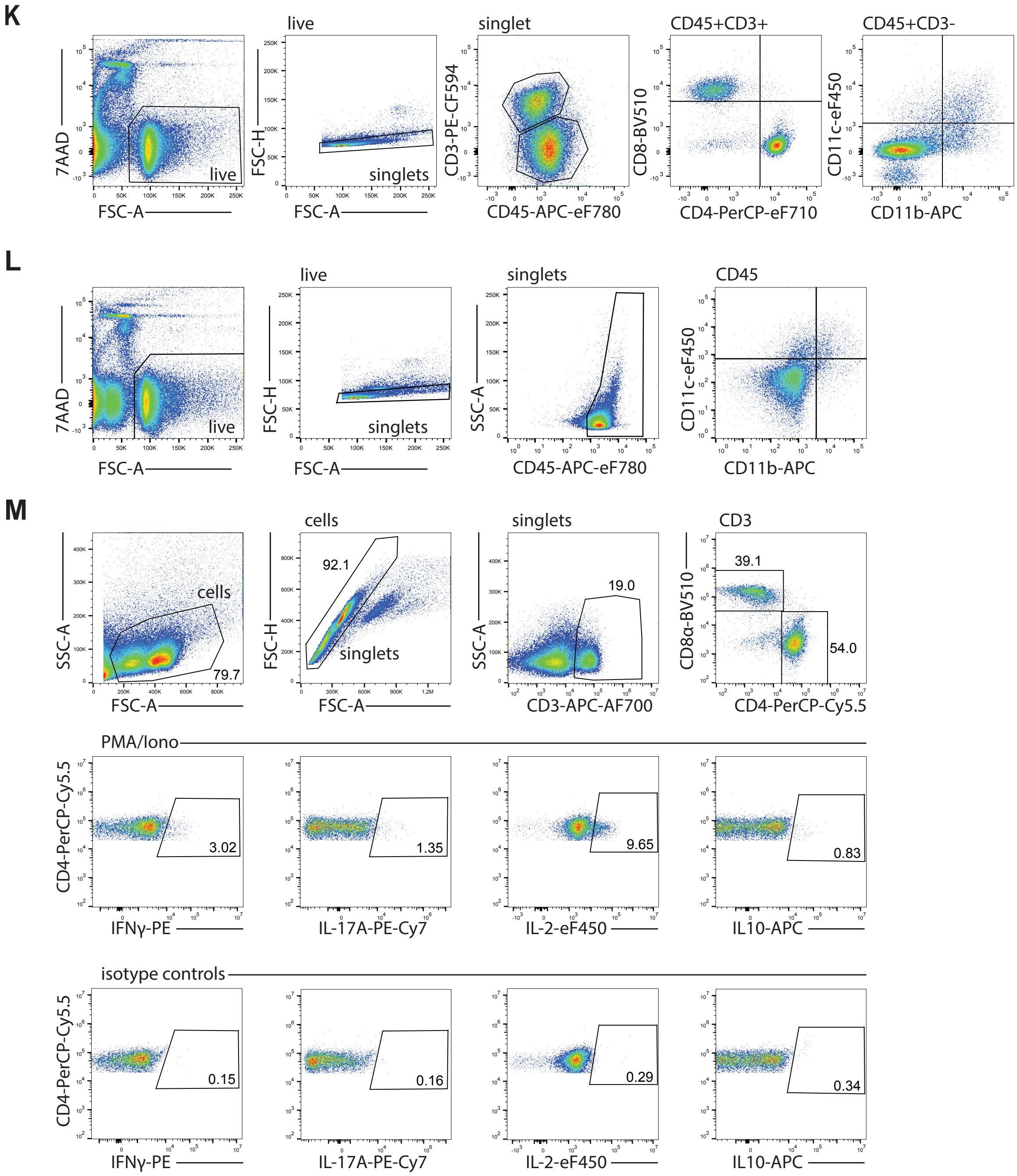

Supplementary Figure 3.

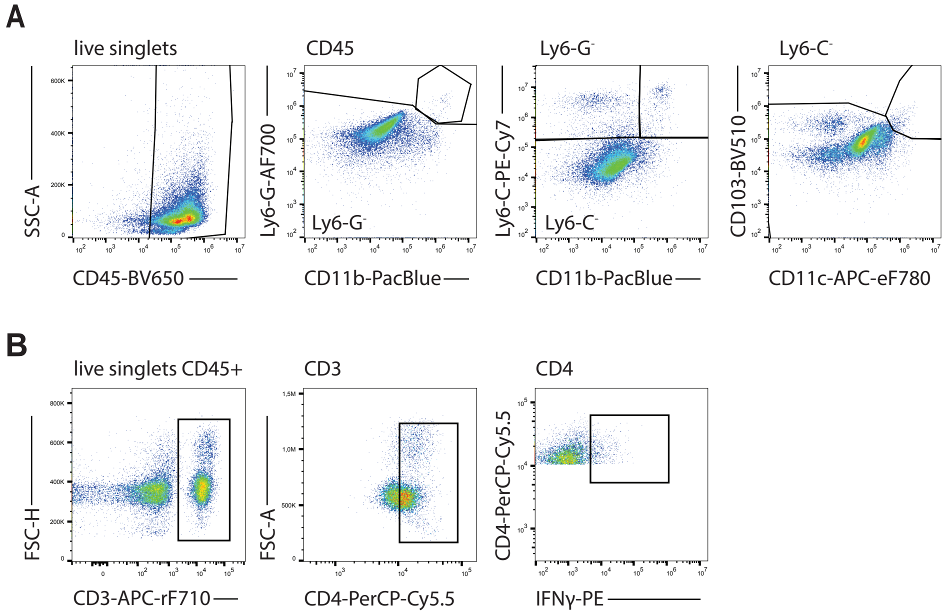

Supplement: Supplementary Figure 1 — (A) Kaplan-Meier-Estimator of survival during AOM/DSS-induced colorectal cancer in male (n=19 per group) and female PTENΔDC mice, (n=8-11 per group) *p<0.05, **p<0.01, log-rank test. (B) serum levels of KC/CXCL1 and IL-33 in PTENΔDC mice measured on day 7 after start of DSS supplementation (n=10-17), ns - not significant, Mann-Whitney test. (C) H&E stainings of FFPE sections of colon swiss rolls from colitic mice on day 7 and day 48, black bars indicate 500µm. (D) serum levels of IL-6 (n=5-6) and KC/CXCL1 (n=6-9) in PTENΔmye measured on day 7 after start of DSS supplementation, ns - not significant, Mann-Whitney test. (E) weight change, (F) serum levels of IL-6, and (G) colon length during acute DSS-induced colitis after DC transfer until day 10 (n=6 per grp. [file DataSheet_1.pdf]
